# Five cases of new‐onset pemphigus following vaccinations against coronavirus disease 2019

**DOI:** 10.1111/1346-8138.16554

**Published:** 2022-08-17

**Authors:** Alberto Corrá, Francesca Barei, Giovanni Genovese, Martina Zussino, Cristina B. Spigariolo, Elena B. Mariotti, Lavinia Quintarelli, Alice Verdelli, Marzia Caproni, Angelo V. Marzano

**Affiliations:** ^1^ Section of Dermatology, Department of Health Science University of Florence ‐ Piero Palagi Hospital Florence Italy; ^2^ Unit of Dermatology Fondazione IRCCS Ca’ Granda Ospedale Maggiore Policlinico Milan Italy; ^3^ Department of Pathophysiology and transplantation University of Milan Milan Italy; ^4^ Rare Skin Diseases Unit, Azienda USL Toscana Centro, ERN‐SKIN member, Department of Health Sciences University of Florence Florence Italy

**Keywords:** COVID‐19, mRNA‐1273, mRNA BNT162b2, pemphigus foliaceus, pemphigus vulgaris

## Abstract

Pemphigus is a group of blistering disorders characterized by the formation of intraepithelial blisters in skin and mucous membranes induced by the binding of circulating autoantibodies to intercellular adhesion molecules. The pathogenesis is complex and not fully understood; however, genetic predisposition and various triggers are widely accepted as key factors in pemphigus development. A few cases of new‐onset pemphigus following coronavirus disease 2019 (COVID‐19) vaccination have already been published. The present paper reports a total of two cases of pemphigus foliaceous and three cases of pemphigus vulgaris that occurred following vaccinations against COVID‐19, with anamnestic, clinical, and diagnostic data collection suggesting assumptions over a possible causal correlation.

## 1 INTRODUCTION

Pemphigus is a group of blistering disorders characterized by acantholysis, resulting in the formation of intraepithelial blisters in mucous membranes and skin. The process of acantholysis is induced by the binding of circulating autoantibodies to intercellular adhesion molecules (mainly desmoglein 1 and desmoglein 3). A few cases of new‐onset pemphigus following coronavirus disease 2019 (COVID‐19) vaccination have already been published. Herein, we present five cases, summarized in Tables 1 and 2, of new‐onset pemphigus following vaccinations against COVID‐19. Specifically, we describe two cases of pemphigus foliaceus (PF) and three cases of pemphigus vulgaris (PV).

**TABLE 1:** Demographics and clinical features of reported cases

Case	Age/sex	Type of pemphigus	Latency	COVID‐19 vaccine before the clinical onset	Previous COVID‐19 vaccination	COVID‐19 vaccine after the clinical onset	Anamnesis	Concomitant medication	Treatments
1	61/woman	PV	3 d	mRNA BNT162b2 (COVID‐19 vaccine booster dose)	Two doses of mRNA‐1273 (well tolerated)	None	Arterial hypertension, undifferentiated connective tissue disease	Prednisone, acetylsalicylic acid, amlodipine, methotrexate	Prednisone 25 mg/d for 4 wk then slowly tapered to 6.25 mg/d in 17 wk Current treatment: prednisone 6.25 mg/d
2	80/man	PF	17 d	mRNA BNT162b2 (COVID‐19 vaccine booster dose)	Two doses of mRNA BNT162b2 (well tolerated)	None	Arterial hypertension, gastroesophageal reflux	Amiloride, hydrochlorothiazide, esomeprazole	Prednisone 50 mg/d for 2 wk, then tapered to 31.5 mg/d in 9 wk Following the lack of improvement, a cycle of rituximab was administered (two doses of 1 g each 15 d apart). Three weeks later, mycophenolate was added Current treatment: mycophenolate 2 gr/d and prednisone 12.5 mg/d
3	66/woman	PF	4 wk	mRNA BNT162b2 (second dose COVID‐19 vaccine)	One dose of mRNA BNT162b2 (well tolerated)	mRNA BNT162b2 (without clinical worsening)	Arterial hypertension, chronic coronary artery disease, gastroesophageal reflux, hypercholesterolemia	Rabeprazole, ticlopidine, atorvastatin, amlodipine, hydrochlorothiazide	Prednisone 43.75 mg/d for 2 wk, then slowly tapered to 5 mg/d in 13 wk Because of clinical worsening, mycophenolate was added but discontinued after only 2 wk for secondary effects. Thus, prednisone was increased to 10 mg/d, then tapered to 5 mg/d in 8 wk Current treatment: prednisone 5 mg/d
4	73/woman	PV	4 wk	mRNA BNT162b2 (COVID‐19 vaccine booster dose)	Two doses of mRNA BNT162b2 (well tolerated)	None	Osteoporosis, major depression	Alendronate, vitamin D supplementation, haloperidol	Prednisone 37.5 mg/d for 3 wk, then 25 mg/d for 5 wk. Rituximab (two infusion of 1 g each separated by 15 d) was added and prednisone was increased to 37.5 mg/d again Current treatment: prednisone 18.5 mg/d
5	63/woman	PV	4 wk	Vaxzevria (ChAdOx1) nCoV‐19 vaccine (first dose)	None	Vaxzevria (ChAdOx1) nCoV‐19 (with clinical worsening)	Arterial hypertension	Zofenopril, hydrochlorothiazide, pantoprazole	Treatment with prednisone 1 mg/kg per d was started along with a total of 2 g of rituximab injection, with rapid improvement of mucocutaneous lesions and partial remission in 8 wk

Abbreviations: COVID‐19, coronavirus disease 2019; mRNA, messenger RNA; PF, pemphigus foliaceous; PV, pemphigus vulgaris.

**TABLE 2:** Immunopathological features of reported cases

Case	Histopathology	DIF	IIF	ELISA	Diagnosis
1	Suprabasal intraepithelial cleavage with acantholysis	Intercellular IgG/C3 deposits	Intercellular IgG deposits	Anti‐DSG1 106 IU/mL (n.v. < 14) Anti‐DSG3 15.5 IU/mL (n.v. < 7)	PV
2	Subcorneal acantholysis with neutrophilic infiltration within the blister	Negative	Intercellular IgG deposits	Anti‐DSG1 149 IU/mL (n.v. < 14)	PF
3	Subcorneal acantholysis with neutrophils within the blister	Intercellular IgG deposits	Negative	Negative	PF
4	Erosive mucositis with diffuse acantholytic cells	Intercellular IgG/C3 deposits	Intercellular IgG deposits	Anti‐DSG3 70 IU/mL (n.v. < 7)	PV
5	Intraepithelial cleavage with acantholysis	Intercellular IgG/C3 deposits	Intercellular IgG deposits	Anti‐DSG1 62.2 IU/mL (n.v. < 14) Anti‐DSG3 157.3 IU/mL (n.v. < 7)	PV

Abbreviations: DIF, direct immunofluorescence; DSG1, desmoglein 1; DSG3, desmoglein 3; ELISA, enzyme‐linked immunosorbent assay; IIF, indirect immunofluorescence; n.v. normal value; PF, pemphigus foliaceous; PV, pemphigus vulgaris.

## 2 CASE REPORT

### 2.1 Case 1

**FIGURE 1:**
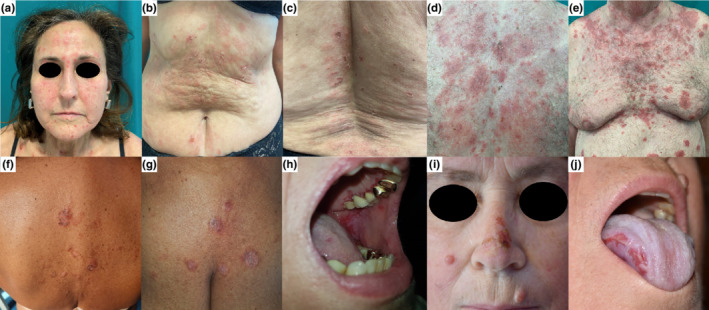
Clinical pictures of our cases. Case 1: scattered superficial blisters and erythematous patches with scaly erosions involving the face and lower trunk (a–c). Case 2: diffuse erythematous‐squamous patches with scaly and crusted erosions involving the face and the trunk, following a seborrheic distribution (d, e). Case 3: a few erosive lesions, diffuse scales, and crusted erosions involving the trunk (f, g). Case 4: painful erosions on the gums and soft palate (h). Case 5: erosive lesions on the oral cavity, nose, right cheek, and abdomen (i, j).

A 61‐year‐old White woman presented to our institution for the appearance of a cutaneous rash 3 days after the COVID‐19 vaccination booster dose with the messenger RNA (mRNA) BNT162b2 (Comirnaty, Pfizer‐BioNTech) vaccine. She had received two previous doses of mRNA‐1273 (Moderna) vaccine 8 and 6 months earlier, with both being well tolerated. The patient had arterial hypertension and undifferentiated connective tissue disease for which she was under chronic therapy with prednisone 2.5 mg/d, acetylsalicylic acid 100 mg/d, amlodipine 5 mg/d, and methotrexate 15 mg/wk (temporarily discontinued for vaccine injections). The patient denied any history of skin disease prior to vaccination. Dermatological examination revealed a few scattered superficial blisters and erythematous patches with scaly erosions involving the face and lower trunk (Figure 1a–c). No oral lesions were seen. Histopathological examination showed suprabasal intraepithelial cleavage with acantholysis. Direct immunofluorescence (DIF) demonstrated intercellular deposition of IgG and C3. Indirect immunofluorescence (IIF) showed intercellular IgG deposits. Enzyme‐linked immunosorbent assay (ELISA) was positive for anti–desmoglein 1 (anti‐DSG1) (106 IU/mL) and anti–desmoglein 3 (anti‐DSG3) antibodies (15.5 IU/mL). A diagnosis of PV was made.

### 2.2 Case 2

An 80‐year‐old White man developed an acute cutaneous eruption 17 days after his booster dose with the BNT162b2 vaccine. The patient had his first two doses 8 months (21 days apart) before the booster, which were both well tolerated. The patient had arterial hypertension and gastroesophageal reflux, for which he was under chronic therapy with amiloride, hydrochlorothiazide, and esomeprazole. Physical examination revealed diffuse erythematous‐squamous patches with scaly and crusted erosions involving the face and the trunk, following a seborrheic distribution (Figure 1d,e). Histopathological examination revealed subcorneal acantholysis with neutrophilic infiltration within the blister. DIF was negative, while IIF showed intercellular IgG deposits. ELISA was positive for anti‐DSG1 antibodies (149 IU/mL), allowing for the diagnosis of PF.

### 2.3 Case 3

A 66‐year‐old White woman complained of a cutaneous eruption that appeared 4 weeks after her second dose of BNT162b2 vaccine. She had arterial hypertension, chronic coronary artery disease, gastroesophageal reflux, and hypercholesterolemia, for which she was taking treatment with rabeprazole, ticlopidine, atorvastatin, amlodipine, and hydrochlorothiazide. Physical examination revealed few erosive lesions, diffuse scales, and crusted erosions involving the trunk (Figure 1f,g). Histopathological examination revealed subcorneal acantholysis with neutrophils within the blister. DIF demonstrated intercellular deposition of IgG. IIF and ELISA were both negative. A diagnosis of PF was made.

### 2.4 Case 4

A 73‐year‐old White woman came to our attention for severe oral pain that occurred about 1 month after the booster dose of the BNT162b2 vaccine. The patient had received her first two doses of BNT162b2 vaccine 6 and 5 months earlier, which were both well tolerated. The patient had osteoporosis and senile dementia, for which he was under chronic therapy with alendronate, vitamin D supplementation, and haloperidol. Physical examination revealed painful erosions on the gums and soft palate (Figure 1h). No cutaneous lesions were observed. Histopathological examination showed erosive mucositis with diffuse acantholytic cells. DIF showed intercellular deposition of IgG and C3. IIF showed intercellular IgG deposits. ELISA was positive for anti‐DSG3 antibodies (70 IU/mL). A diagnosis of PV was made.

### 2.5 Case 5

A 63‐year‐old White woman presented with painful oral erosions involving mainly the oral cavity and the skin of the face and trunk. Patients' medical history was unremarkable, except for arterial hypertension controlled with systemic therapy. She complained of three small erosions that appeared about 4 weeks after the first dose of Vaxzevria (ChAdOx1) nCoV‐19 vaccine (Oxford‐AstraZeneca), localized respectively on the floor of the mouth, soft palate, and right side of the buccal mucosa. Successively, she received the second vaccine dose 12 weeks after the first, experiencing a worsening of the disease. In fact, 4 days after vaccine injection, new erosions appeared on the floor of the mouth, tongue, and gums, while cutaneous erosions affected the nose, right cheek, and abdomen (Figure 1i,j). The patient therefore underwent a skin biopsy, which revealed acantholysis on histology and intercellular deposition of IgG and C3 on DIF. Serological investigation supported the diagnosis of PV showing intercellular deposition of IgG and positive staining for anti‐DSG1 and anti‐DSG3. ELISA was positive for anti‐DSG1 and anti‐DSG3 antibodies, with titers of 62.2 IU/mL and 157.3 IU/mL, respectively.

## 3 DISCUSSION

Several cases of new‐onset pemphigus following the anti–COVID‐19 vaccine have already been published. A review by Calabria et al reported cases of severe autoimmune blistering diseases (AIBDs) associated with COVID‐19 vaccination: they found six cases of PV and one case of PF. Coherently with our report, the median age of the cases of the pemphigus group was 60 years (interquartile range, 50–76 years) with no sex predilection (four women, three men). Among all of the cases of AIBDs, the majority (22 [62.9%]) developed after BNT162b2 vaccine administration, six (17.1%) after mRNA‐1273 administration, three (8.6%) after ChAdOx1 administration, and three (8.6%) after CoronaVac administration.^1^ According to Calabria et al's study and other studies in the literature, the Comirnaty vaccine seems to present the higher rate of association with onset of AIBDs, but this may probably be explained by the wider use of the BNT162b2 vaccine compared with the others in Western populations.^2^ In a recent paper describing autoimmune skin disease flares following COVID‐19 vaccine, full immunization with mRNA‐1273 showed a higher likelihood of association with worsening of preexistent disease than BNT162b2, but the difference was not significant.^3^

The pathogenesis of autoimmune disorders following antiviral vaccinations is still under debate. One of the suggested mechanisms is immune cross‐reactivity caused by molecular mimicry.^4^, ^5^ In fact, this phenomenon was advocated to explain several cases of pemphigus developed following exposure to bacterial and viral antigens, as well as pharmacological agents.^6^ However, Kasperkiewicz et al.^7^ failed to find cross‐reactivity between circulating anti–severe acute respiratory syndrome coronavirus 2 (SARS‐CoV‐2) antibodies and pemphigus or pemphigoid autoantigens in a 24‐patient series. A relationship between autoimmune bullous diseases and COVID‐19 vaccination has been hypothesized by other authors,^8^ suggesting a transient immune activation in predisposed individuals with subclinical autoimmunity. The hypothesis of a nonspecific, nonselective mechanism of action may better explain the wide variety of immune‐mediated diseases described following COVID‐19 immunization, ranging from autoinflammatory to mixed or autoimmune manifestations, which may both be multisystemic or organ‐specific such as pemphigus. Moreover, the onset of immune‐mediated diseases seems to occur more frequently in individuals with a preexisting rheumatic or autoimmune background, thus supporting the importance of individual predisposition.^9^ The role of personal susceptibility is even more supported by the fact that autoimmune bullous diseases with higher incidence in their idiopathic form, such as bullous pemphigoid, also seem to be more frequently triggered or boosted by COVID‐19 vaccines.^10^ In addition, Case 1 suggests that chronic immunosuppressive therapies prescribed for other concomitant diseases might maintain the autoimmune bullous disorder in a latent or subclinical stage until the balance is broken by strong precipitating factors such as vaccinations.

An innate inflammatory process is triggered by both BNT162b2 and ChAdOx1 vaccines caused by adjuvant function of mRNA itself and adenoviral proteins, respectively, which binds to different toll‐like receptors, enhancing the production of type I interferon and several proinflammatory cytokines.^11^ Type I interferon is well‐known for its role in stimulating antigen presentation, B‐cell differentiation, and IgG secretion.^12^, ^13^, ^14^ However, a clear link between the vaccine's mechanism of action and pemphigus development is still far from being elucidated. In our case series, the timing of pemphigus onset is the only element that may suggest a causal relationship. The presence of a correlation between these events should be supported by larger population‐based studies investigating an incidence variation of pemphigus and other autoimmune bullous diseases before and after large‐scale vaccine administration. Until those kinds of studies are available in the literature, physicians should be aware of the possibility of autoimmune blistering diseases following SARS‐CoV‐2 vaccine, with the possibility of a causal relationship.

## CONFLICT OF INTEREST

The authors declare no conflicts of interest.
